# Questionnaire Survey of Burnout Amongst Dentists in Singapore

**DOI:** 10.1016/j.identj.2021.08.054

**Published:** 2021-10-01

**Authors:** Surinder Arora, Alec Knight

**Affiliations:** School of Population Health and Environmental Sciences, Faculty of Life Sciences and Medicine, King's College London, London, United Kingdom

**Keywords:** Burnout, Work stress, Psychosocial work environment, Exhaustion, Fatigue

## Abstract

**Background:**

Burnout results from ongoing, unsuccessfully managed workplace stress, resulting in feelings of exhaustion, increased mental distance from one's job, and reduced professional efficacy.

**Method:**

This research used a cross-sectional questionnaire survey design. Graduated dentists in Singapore completed an online questionnaire comprising 5 sections: (a) demographics (3 items); (b) working conditions and experience (12 items); (c) the Copenhagen Burnout Inventory (CBI) (19 items); (d) supplementary questions assessing causes and outcomes of stress and burnout (15 items); and (e) other outcomes (3 items).

**Results:**

Overall, low to moderate levels of burnout were reported by the 210 survey respondents. Average CBI scale scores (out of 100) were as follows: personal burnout = 49.14, work-related burnout = 46.41, and patient-related burnout = 37.72. High to severe levels of burnout were self-reported by 24 individuals (11.3%) for personal burnout, 17 individuals (8.0%) for work-related burnout, and 9 individuals (4.2%) for patient-related burnout.

**Conclusions:**

Levels of burnout were generally low to moderate in this sample, with a small proportion of dentists experiencing high levels of burnout. Further research is required to gain clarity on stress and burnout levels across different occupational designations and dentistry settings in Singapore.

## Introduction

Burnout was recently designated an occupational phenomenon in the International Classification of Diseases,^1^ although it is not recognised as a medical condition.^2^ It is caused by ongoing, unsuccessfully managed workplace stress, resulting exhaustion, increased mental distance from work, and reduced professional efficacy.^1^ Research on burnout is increasing with burnout particularly recognised as an occupational hazard in people-oriented professions.^3^

According to the Ministry of Health (MOH), Singapore had 2292 practising dentists in 2018.^4^ High levels of stress and suicide are reported in dentistry,^5^ with burnout leading to “diminished professional standards.”^6^ Earlier research suggested that dentists are prone to burnout, anxiety disorders, and clinical depression.^7^ In addition, work engagement has been associated with dentists’ clinical productivity and remuneration.^8^ Poor quality of care and patient safety may be consequences of burnout,^9^ which may be associated with “suboptimal patient care” amongst physicians.^10^ Burnout in clinicians may result in undesirable behaviours, including inaccurate record keeping, failure to discuss treatment options or answer queries fully,^11^ and errors in treatment and medications.^12^

Numerous burnout risk factors have been identified amongst dentists, including time, scheduling, and business pressures; staffing; patient treatments and expectations; and remuneration.^13^ Seeing more than 30 patients per day may also contribute to burnout.^14^ Reported burnout prevalence amongst dentists varies widely, from 2% to 3% in Spain^15^ to 88% in the UK.^16^ There is also variability in the prevalence of burnout amongst different subgroups including oral surgeons,^17^ teaching dentists,^18^ and other dental groups.^16^

Research has reported considerable variability in the prevalence of dentists’ burnout internationally. Only 7% of dentists in Hong Kong reported high levels of burnout.^19^ However, roughly half of Korean dentists scored high on burnout subscales,^20^ whilst 47.8% of young Indian dentists experienced high levels of burnout.^21^ Forty percent of Greek dental students were found to be at risk for burnout.^22^ Similarly, 7.4% of American dentists were significantly affected by burnout, with 83% reporting dentistry to be very stressful.^23^ A quarter of dentists in a UK study had serious burnout risk.^24^ In contrast, 93.3% of Malaysian dentists reported being “generally happy.”^25^ A study in India reported low burnout levels amongst doctors and dentists,^26^ whilst a Swiss study reported low burnout levels amongst dental residents.^27^ Military dentists in the United States also reported low burnout levels.^28^ Such findings indicate a range of burnout prevalence across different localities, warranting different management approaches.

To date, there has been no burnout research on dentists in Singapore and limited evidence on burnout amongst Singaporean health care professionals generally. Health care groups studied include physicians,^29^ nurses,^30^ and mental health professionals.^31^ A clear research gap exists regarding Singaporean dentist burnout in Singapore. Our primary research objective was to establish burnout prevalence and severity amongst dentists in Singapore. Secondary objectives included examining burnout associations with sex, designation (general dentist, specialist, or other), age, and work experience as well as self**-**perceived causes and outcomes of stress. Supplementary aims included gathering suggestions for management of stress and establishing the reliability of the Copenhagen Burnout Inventory (CBI) amongst dentists in Singapore.

## Methods

### Burnout questionnaire scales

The Maslach Burnout Inventory (MBI) for Medical Personnel is the leading instrument for assessing burnout amongst clinicians.^32^ Despite its widespread use, the MBI is a commercial product that has been criticised on numerous grounds.^33^ Notably, MBI subscales “depersonalization” and “reduced personal accomplishment” have been characterised as coping strategies rather than components of burnout. In response, researchers developed the CBI, which has established validity and reliability and is a popular, public-domain burnout inventory.^33^ It incorporates recent research developments, with items focusing on individuals, working environments, and patients.^34^ The CBI has good psychometric properties, with research on Australian dentists suggesting that it is at least equivalent to the MBI burnout assessment.^35^

### Measures and procedures

A cross-sectional questionnaire survey was conducted. Graduated dentists working in Singapore completed our survey via the online platform Typeform (Typeform S.L.). Our questionnaire comprised 5 sections: (a) demographic information (3 items); (b) working conditions and experience (12 items); (c) CBI (19 items); (d) supplementary questions assessing causes and outcomes of stress (15 items); and (e) other outcomes (3 items).

The CBI comprises 3 scales: (a) personal burnout (6 items); (b) work-related burnout (7 items); and (c) patient-related burnout (6 items). Each scale is considered a continuous variable. Participants responded using 5-point Likert scales with the following labels: 5 = “to a very high degree,” 4 = “to a high degree,” 3 = “somewhat,” 2 = “to a low degree,” and 1 = “to a very low degree” (for scales 1 and 3) and 5 = “always,” or 4 = “often,” 3 = “sometimes,” 2 = “seldom,” and 1 = “never/almost never” (for scale 2). Participants were classified as nonresponders with fewer than 3 completed items (for scales 1 and 3) or 4 completed items (scale 2), per scale guidelines. Evidence for the validity of the CBI in assessing dentist burnout has been provided by studies on dentists in India,^21^ Australia,^35^ and Brazil.^36^

Data were collected from January 2 to March 3, 2020. Earlier, the survey was piloted with 4 dentists to gain feedback. A link to the survey was emailed via the Singapore Dental Association (SDA) mailing list 3 times (January 3, January 20, and February 10, 2020). A link to the questionnaire was also posted on the first author's social media accounts and was shared on a closed professional dental group. An opt-in incentive (hotel stay) was offered for survey completion. The prize was sponsored by Nuffield Dental Holdings and MC Ceramics Singapore, which did not have access to data and had no influence on the study. Data were stored on an encrypted, password-protected memory stick to which only the research team had access.

### Statistical analyses

All data analyses were conducted using IBM SPSS Statistics for Macintosh, Version 27 (IBM Corp.). Cronbach's alpha (α) was used to assess the internal consistency of CBI scales. One-way analysis of variance (ANOVA) was used to examine group differences in burnout (sex, designation, and years of experience). Spearman's rank correlation was used to examine associations between burnout and stress risk factors and outcomes.

### Ethics

Minimal Risk Ethical Approval was provided by the ethics committee of King's College London (MRSU-19/20-14859), which was the institutional affiliation of both authors during the study. Informed consent was obtained from all participants, and survey responses were anonymous. Participation was voluntary and participants were free to withdraw at any time. General Data Protection Regulation (GDPR) guidelines were adhered to.^37^ As stigma is associated with mental health issues, burnout may be perceived as a sensitive topic.^38^ Therefore, contact details for the Institute of Mental Health were provided for those requiring further support.

## Results

In all, 216 of 2293 dentists responded (9.4%), and 210 provided complete responses (9.2%).

### Demographics

Demographic characteristics of the sample are displayed in Table 1.

**Table 1:** Demographic and basic work characteristics of survey respondents.

	%	n
Sex		
Female	58.6	126
Male	40.9	88
Undisclosed	0.5	1
Age (years)		
23-29	33.3	72
30-39	30.1	65
40-49	17.1	37
50-59	10.7	23
60 and older	8.8	19
Ethnicity		
Chinese	87.0	187
Indian	7.4	16
Malay	1.0	2
Caucasian	2.3	5
Other	2.0	4
Job role (designation)		
General dentist	87.0	187
Specialist	10.7	23
Other	2.3	5

### CBI item and scale responses

Data are reported in raw percentage form (Likert values 1, 2, 3, 4, or 5; see Table 2). When asked “Do you feel burnt out because of your work?”, 25% of respondents (n = 56) reported to a “high” or “very high degree,” 34% of respondents (n = 72) reported “somewhat,” and 39% of respondents (n = 84) reported to a “low” or “very low degree.”

**Table 2:** Item-level responses for CBI scales (raw score percentages).

Likert scale value	1	2	3	4	5	Total number of responses	Missing responses
CBI scale 1: personal burnout items							
1	0.9	4.7	37.2	40.5	14.4	210	6
2	2.8	17.2	40.0	29.3	8.4	210	6
3	3.7	24.2	35.8	24.2	10.2	211	5
4	26.0	33.5	28.4	7.9	2.8	212	4
5	4.2	27.9	34.9	25.1	6.0	211	5
6	16.7	38.1	31.2	9.3	3.3	212	4
CBI scale 2: work-related burnout items							
1	6.1	29.2	37.7	19.3	7.5	212	4
2	11.3	28.3	34.0	20.8	5.7	212	4
3	11.8	35.8	40.6	6.1	5.7	212	4
4	8.5	21.7	35.4	25.9	8.5	212	4
5	14.2	25.9	34.0	15.6	10.4	212	4
6	18.4	35.8	30.7	12.7	2.4	212	4
7	4.2	14.6	33.5	35.8	11.8	212	4
CBI scale 3: patient-related burnout items							
1	15.6	50.0	26.4	3.8	3.3	210	6
2	18.4	49.1	25.9	3.8	2.8	212	4
3	14.2	33.5	36.8	10.8	4.2	211	5
4	10.8	31.6	30.7	18.4	8.5	212	4
5	18.9	32.1	40.1	4.7	4.2	212	4
6	21.7	25.9	25.5	17.5	8.5	210	6

Likert scale values for column headings are as follows.

1 = always/to a very high degree; 2 = often/to a high degree; 3 = sometimes/somewhat; 4 = seldom/to a low degree; 5 = never or almost never/to a very low degree. Missing n indicated number of nonrespondents for each question.

Totals do not sum to 100 due to rounding to one decimal place.

CBI = Copenhagen Burnout Inventory.

Research has indicated that internal consistency ranging from 0.70 to 0.95 is “good.”^39^ As such, scale alpha values for CBI scales 1, 2, and 3 were considered acceptable. Per convention in CBI research, means, standard deviations, and minimum and maximum values are reported (see Table 3). The mean score for patient-related burnout was lower than for the other 2 scales.

**Table 3:** Scale-level descriptive characteristics of participant responses.

Scale	Mean	SD	Minimum	Maximum	α
CBI scale 1: personal burnout	49.14	19.73	32.21	66.35	0.895
CBI scale 2: work-related burnout	46.41	17.28	36.20	59.08	0.784
CBI scale 3: patient-related burnout	37.72	20.55	31.16	45.78	0.884

Per convention in research employing the CBI, original values were recoded into scale scores out of 100. Scoring was as follows: always/to a very high degree: 100; often/to a high degree: 75; sometimes/somewhat: 50; seldom/to a low degree: 25; never/almost never/to a very low degree: 0.

CBI = Copenhagen Burnout Inventory.

Histograms were used to examine data spread for each CBI scale. Standardised score cutoffs were used: low (<50), moderate (50-74), high (75-99), and severe (100).^40^ Twenty-four individuals (11.3%) scored more than 75 points on CBI scale 1, 17 individuals (8.0%) on CBI scale 2, and 9 individuals (4.2%) on CBI scale 3. Although mean figures indicated low to moderate burnout, a proportion of the sample reported high and severe burnout.

### Group differences in burnout for sex, designation, and years of experience

One-way ANOVA was conducted to compare burnout scale scores for males and females. The difference between the groups was nonsignificant (Table 4). Another one-way ANOVA was conducted to compare burnout scale scores between dentist designations (ie, dentist, specialist, or other). The “other” category comprised 1 registrar and 4 residents in training, so we exercised caution in interpreting findings about this group. Group means indicated that specialists reported the lowest burnout levels (CBI scale 1 = 44.84, CBI scale 2 = 40.65, CBI scale 3 = 27.38), whilst the “other” category displayed moderate burnout levels for CBI scales 1 and 2 (69.2 and 57.1, respectively). There were no statistically significant differences amongst designations. A Tukey post hoc test was used to analyse the direction of designation group differences for CBI scale 1. “Specialist” and “other” groups differed significantly (*p* < .035), with “specialist” (44.84) scoring lower than “other” (69.17). Other group differences approaching statistical significance were between “general dentists” and “other” for CBI scale 1 (*p* = .054) and between “general dentists” and “specialists” for CBI scale 3 (*p* = .057).

**Table 4:** CBI scale scores and standard deviations for different dentist sexes, designations, years of experience, age groups, and numbers of patients seen per day.

	CBI 1: personal burnout	CBI 2: work-related burnout	CBI 3: patient-related burnout	n
Sex				
Male	51.27 (22.10)	48.19 (18.11)	39.85 (22.15)	85
Female	47.33 (17.43)	45.57 (16.53)	36.00 (19.19)	125
Designation				
General dentist	48.51 (19.02)	46.76 (17.36)	38.20 (20.31)	185
Specialist	44.84 (22.70)	40.65 (15.71)	27.38 (17.46)	21
Other	69.17 (26.45)	57.14 (18.39)	48.33 (32.49)	5
Years of experience				
Less than 1 year	45.83 (9.77)	49.60 (10.17)	45.37 (19.37)	9
1-5 years	52.03 (19.20)	50.43 (17.36)	40.20 (21.36)	74
6-10 years	48.58 (20.13)	46.52 (19.11)	38.11 (22.29)	41
10 years or more	46.07 (20.54)	42.65 (16.28)	33.76 (18.74)	88
Age group, years				
23-29	51.88 (16.62)	51.76 (15.29)	40.79 (19.25)	71
30-39	50.00 (21.99)	46.43 (19.17)	38.87 (23.38)	64
40-49	46.53 (21.54)	42.96 (18.18)	33.45 (20.18)	36
50-59	40.40 (21.19)	38.82 (16.52)	34.06 (18.36)	23
60 and older	45.60 (14.25)	41.87 (10.16)	30.32 (16.03)	18
Number of patients seen per day				
1 = very low	56.67 (24.31)	53.93 (25.34)	51.25 (30.37)	10
2 = low	44.51 (18.34)	42.99 (16.65)	36.81 (20.71)	79
3 = intermediate	49.77 (18.49)	47.97 (16.45)	36.86 (19.11)	72
4 = high	52.72 (21.92)	47.94 (18.30)	38.30 (22.30)	26
5 = very high	52.95 (19.72)	49.55 (14.83)	35.24 (15.44)	24

CBI = Copenhagen Burnout Inventory.

Spearman's rank coefficient was used to examine the association between years of experience and CBI scale scores. A weak negative association between experience and burnout was found for work-related burnout (n = 212; *r* = –0.192; *p* < .005) and patient-related burnout (n = 212; *r* = –0.129; *p* < .061). The group with highest work-related burnout was those with 1 to 5 years of experience (mean = 50.4), followed in order by those with less than 1 year experience, 5 to 10 years of experience, and more than 10 years of experience (mean = 42.6).

### Burnout and age

Mean burnout scale scores were examined for the different age groups. There was generally an inverse association between age and CBI scales 1 and 2 (personal and work-related burnout): Older age groups tended to report lower levels of burnout. However, the exception was dentists aged 60 and older, for whom reported burnout levels were higher. Patient-related burnout was highest for dentists aged 23 to 29, then in order dentists aged 30 to 39, 50 to 59, 40 to 49, and 60 and older (see Table 4).

### Burnout and number of patients seen per day

The association between “number of patients seen per day” and burnout was not statistically significant. This was examined by calculating CBI scale means and standard deviations for 5 groups (1-5 patients = very low; 6-10 patients = low; 11-15 patients = intermediate; 16-20 patients = high; 21 or more patients = very high). Respondents in groups 1 (56.67) and 5 (52.95) indicated the highest levels of personal burnout, with CBI scale scores generally highest for dentists with very low or very high numbers of patients. Dentists with low, intermediate, and high numbers of patients tended to report lower burnout across all CBI scales (see Table 4).

### Burnout and the risk factors and outcomes of stress

Items assessing risk factors for stress and outcomes of stress were analysed against CBI scale scores using Spearman's rank correlation. There were numerous significant associations between burnout and risk factors (see Table 5). “Pressures associated with treating patients” was highly correlated with all CBI scales (*p* < .01). “Feeling guilty about how a patient was treated” was also highly correlated with patient-related burnout (*p* < .01).

**Table 5:** Spearman's rank correlation coefficients for CBI scale scores and risk factors/outcomes of stress items.

	CBI 1: personal burnout	CBI 2: work-related burnout	CBI 3: patient-related burnout
**Risk factors for stress**			
Time and scheduling pressures	.320⁎⁎	.326⁎⁎	.189⁎⁎
Staffing issues	.166*	.155*	0.062
Patient/public perception of you	.330⁎⁎	.373⁎⁎	.318⁎⁎
Professional concerns (CPE/fear of litigation/making mistakes)	.349⁎⁎	.390⁎⁎	.383⁎⁎
Pressures associated with treating patients	.448⁎⁎	.513⁎⁎	.496⁎⁎
Business pressures	.218⁎⁎	.223⁎⁎	.122
Patient expectations	.352⁎⁎	.381⁎⁎	.407⁎⁎
Pay-related pressures	.335⁎⁎	.407⁎⁎	.363⁎⁎
Striving for perfection	.340⁎⁎	.369⁎⁎	.280⁎⁎
**Outcomes of stress**			
Not fully discussing treatment options or answering questions	.213⁎⁎	.231⁎⁎	.287⁎⁎
Making treatment or medication errors	.154*	.181⁎⁎	.247⁎⁎
Ignoring how a patient's condition may affect them socially or personally	.198⁎⁎	.256⁎⁎	.298⁎⁎
Feeling guilty about how a patient was treated	.213⁎⁎	.289	.428⁎⁎

⁎ Correlation significant at the .05 level (2-tailed).

⁎⁎ Correlation significant at the .01 level (2-tailed). CBI = Copenhagen Burnout Inventory; CPE = Continuing Professional Education.

### Other stress outcomes and participant suggestions

Nineteen dentists (8.8%) responded “yes” when asked whether they had sought support to cope with stress. Moreover, 60 dentists (27.9%) responded “yes” when asked whether they require further support to manage stress. These are clear indications that there is an unmet need for assistance regarding stress amongst dentists in Singapore.

Our free-text response items were analysed using conventional content analysis.^41^ There were numerous comments for “causes of stress” including physical pain and work–life balance. In relation to “outcomes of stress,” participants indicated that issues included physical ailments, feeling depressed or anxious, loss of appetite, crying in front of patients, thinking about mistakes outside of work, and self-disappointment. When asked what could be done to support dentists, responses included support groups and counselling, yoga and meditation, removing stigma around mental health, more involvement from the SDA and Singapore Dental Council (SDC), and tackling the “unhealthy obsession with working more than those around you.”

## Discussion

The primary research objective was to establish burnout levels amongst dentists in Singapore. In our sample, overall burnout levels were low to moderate, with average scores (out of 100) of 49.14 for personal burnout, 46.41 for work-related burnout, and 37.72 for patient-related burnout. Nevertheless, a proportion of dentists reported high and severe burnout (24 individuals for personal burnout, 17 for work-related burnout, and 9 for patient-related burnout). Our findings suggest lower levels of burnout in dentists in Singapore than amongst resident physicians (80.7%)^29^ and mental health professionals,^31^ who reported average exhaustion and disengagement scores of 62% and 58.5%, respectively. Our findings are closer to the 33.3% prevalence of burnout found in hospital nurses in Singapore.^30^ Nevertheless, research indicates that high levels of burnout may affect patient safety and increase mistakes made at work.^12^ Burnout on CBI scales 1, 2, and 3 were slightly higher in the present study than reported in previous research on dentists employing the CBI.^35^^,^^36^ One other study assessed burnout amongst dentists using the CBI but did not report average scale values, precluding comparison with our findings.

This study found no significant sex difference in burnout subscales. A systematic review of factors contributing to burnout in dentistry indicated that younger age and male sex were associated with higher burnout.^42^ Extant research on the subject is mixed. For example, one study indicated that male dentists reported higher scores on the depersonalisation dimension of the MBI,^43^ whilst another study reported that females aged 26 to 28 years were at highest risk for burnout.^21^ We found no significant difference between dentist designation and burnout, although this finding may have been affected by small group sizes. Nevertheless, mean scores indicated that specialists had the lowest burnout and the “other” group had the highest personal and patient-related burnout. In a study based on a sample of Israeli dentists, specialists experienced lower burnout than general dentists.^44^ It is possible, therefore, that specialisation in a dental field may be associated with low burnout.

There are limited extant findings to support any inverse association between experience and burnout. Notably, the same study in Israel reported a significant negative correlation between burnout and work experience amongst dentists.^44^ Similarly, in the present study a roughly inverse association was reported between personal or work-related burnout and age, with the exception of dentists aged 60 years and older, whose burnout levels were higher. Some dentists may have left the profession or ceased practising by age 60. This survival bias may affect the present findings.^45^ Age, burnout, and retirement amongst dentists is an area warranting further investigation.

Risk factors and outcomes of stress were investigated, and the present results support recent findings.^11^^,^^12^ Risk factors for burnout in dentists include time and scheduling pressures, staffing, patient perceptions and expectations, pressures associated with treating patients, and business- and pay-related stressors.^13^ Seeing more than 30 patients per day is also associated with higher levels of burnout.^14^ Burnout may be linked to undesirable behaviours, including inaccurate record keeping and failure to discuss treatment options or answer queries fully.^11^ There is also a reported association between burnout and making errors during treatment or when prescribing medications.^12^ This research confirmed the psychometric reliability of the CBI.

A strength of this study was that it investigated an important area of policy and research in a novel national context. With numerous scales existing to investigate burnout,^46^ the CBI was selected due to its good validity and reliability, whilst incorporating questions about the individual, work environment, and patients.^34^ However, not all theoretical developments in the burnout sphere are captured in burnout inventories, potentially limiting the validity of the CBI. As the CBI relied on self-reports, response bias may limit the validity of present findings. Those with very high or low burnout may not have responded or may have used this study to highlight their issue. In addition, there may have been central tendency bias relating to the CBI items.^47^ Participants may also have been reluctant to answer honestly due to the sensitivity of the topic and stigma surrounding mental health.^48^ This study indicated low levels of patient-related burnout and moderate levels of personal and work-related burnout. Some respondents reported severe burnout.

The questionnaire was distributed online via the SDA email list. Eighteen percent of dentists are not SDA members and were not contacted directly. Other dentists may not have opened the email or may have chosen not to respond. Low response rates are common in burnout research, with rates ranging from 9% in UK general dentists (n = 22,906)^16^ to 96.3% in military dentists (n = 80).^28^ Although our response rate was low, it was consistent with similar studies. The questionnaire relied on voluntary participation and was initiated at the time of the initial stages of the COVID-19 pandemic, both of which may have affected responses. Since this research has been conducted, COVID-19 has shifted the face of dentistry. New stressors have been introduced that this research did not capture. Nonetheless, the daily aspects of working as a dentist remain, with additional alterations in working requirements to manage COVID-19.

Around 28% of the present sample responded that they required further assistance with burnout. Large-scale research on burnout in dentists is warranted, with significant numbers of dentists reporting severe burnout and requesting support with stress. Future research may help to establish how and why burnout affects dentists in different settings and provide a solid empirical basis for action and policy. Burnout awareness should be addressed by organizations including the SDC, SDA, and MOH. These organisations should consider the development of support mechanisms. Confirming predictors of burnout is an essential step before the formation of support groups and counselling services, as suggested by our respondents. The “Management Standards Approach” suggests that it is the responsibility of employers to protect workers from stress in the workplace.^49^ As many dentists are self-employed, the onus may fall on institutions such as the SDC or MOH. However, this is contingent on these bodies or employers first being aware and second wanting to take action.

## Conclusions

This paper examined burnout levels of dentists in Singapore using a survey including the CBI. The results suggest low to moderate levels of burnout, but a proportion of dentists had high burnout. Further research is required to gain clarity on burnout levels across different dentistry designations and settings in Singapore.

## Appendix Supplementary materials

Image, application 1
