# Supplementary material for: Questionnaire Survey of Burnout Amongst Dentists in Singapore
Source: Int Dent J. 2021 Oct 1;72(2):161–8. doi: 10.1016/j.identj.2021.08.054 (PMC9275171; doi:10.1016/j.identj.2021.08.054)
Supplement: Supplementary file 1 [file mmc1.docx]

**Appendix 1 – Questionnaire**

1. What is your DCR (Dental Council Registration) number? (optional)
Your DCR number will be kept anonymous and will only be used for the prize draw.
 
2. What is your age group?
20-29 years 
30-39 years
40-49 years
50-59 years
60 and above years

3. What is your gender?
Female
Male
Prefer not to disclose
 
4. What is your ethnicity?
Chinese

Indian

Malay

Eurasian

Caucasian

Other (please state)

5. In which country did you receive your undergraduate training?
Singapore 
US
UK
Australia
Hong Kong
Other (please state)

6. In which year did you graduate with your basic dental degree (BDS, DDS or equivalent)?

7. How long have you been working as a dentist in Singapore?

< 1 year

1-5 years

6-10 years

Over 10 years

8. Are you serving a bond? 
Yes
No
 
9. Are you a conditionally registered dentist?
Yes
No
 
10. Are you a
General dentist
Specialist
Other (please specify)

11. Do you work

Full time

Part time

Not working

## Other

12. Which sector do you primarily work in?
 Private sector
 Public sector

Both 
 Other (please specify)

13. Are you currently
a solo practitioner
a partner in a group practice
an associate in private practice
working in a public hospital (e.g. NDC, NUH, KTPH, KKH) 
working in a polyclinic 
working in a military / SAF clinic
(select all that apply)
 
14. What is the nature of your work?
Clinical practice
Teaching 
Research
Other ……please specify
(select all that apply)

 
15. Are you a practice owner or partner with responsibilities outside of clinical work e.g. administration, accounts, HR? 
Yes
No

16. Are the majority of your patients
Paying privately for treatment with their own funds

CHAS (government Community Health Assist Scheme)

Covered by insurance

Other

17. In the last 2 months, on average how many patients have you seen per day? 
Less than 5
6-10
11-15
16-20
21-25
26-30
Over 30

18. CBI scale 1

1. How often do you feel tired?

2. How often are you physically exhausted?

3. How often are you emotionally exhausted?

4. How often do you think: ”I can’t take it anymore”?

5. How often do you feel worn out?

6. How often do you feel weak and susceptible to illness?

Response categories: Always, Often, Sometimes, Seldom, Never/almost never.

Scoring: Always: 100. Often: 75. Sometimes: 50. Seldom: 25. Never/almost never: 0. Total score on the scale

19. How much do the following cause stress in your day to day work?

Response categories: 1 to 5 (1 to a low degree and 5 to a high degree)

## Time and scheduling pressures

## Staffing issues

## Patient/public perception of you

## Professional concerns (CPE/fear of litigation/making mistakes)

## Pressures associated with treating patients

## Business pressures

## Patient expectations

## Pay-related pressures

## Striving for perfection

Other causes (please specify)

20.CBI 2

1. Is your work emotionally exhausting?

2. Do you feel burnt out because of your work?

3. Does your work frustrate you?

4. Do you feel worn out at the end of the working day?

5. Are you exhausted in the morning at the thought of another day at work?

6. Do you feel that every working hour is tiring for you?

7. Do you have enough energy for family and friends during leisure time?

Response categories:

Three first questions: To a very high degree, To a high degree, Somewhat, To a low degree, To a very low

degree.

Last four questions: Always, Often, Sometimes, Seldom, Never/almost never.

(Reversed score for last question).

21. When feeling stressed are you more likely to

Not fully discuss treatment options or answer questions

Make treatment or medication errors

Ignore the social or personal impact of a condition

Feel guilty about how a patient was treated

Other (please specify)

Response categories: 1 to 5 (1 to a low degree and 5 to a high degree)

22. CBI 3

1. Do you find it hard to work with patients?

2. Do you find it frustrating to work with patients?

3. Does it drain your energy to work with patients?

4. Do you feel that you give more than you get back when you work with patients?

5. Are you tired of working with patients?

6. Do you sometimes wonder how long you will be able to continue working with patients?

Response categories:

The four first questions: To a very high degree, To a high degree, Somewhat, To a low degree, To a very low

degree.

The two last questions: Always, Often, Sometimes, Seldom, Never/almost never.

23. What do you think could be done to support dentists with stress or burnout within the profession?

24. Have you sought support with stress or burnout as a dentist?

## Yes/No

25. Do you require further support to manage stress or burnout as a dentist?

## Yes/No

**Appendix 2: Free text responses about other causes of stress, other outcomes of stress and suggestions for supporting dentists with stress and burnout**

|  |  |
| --- | --- |
| \| **Other causes of stress (Q19)** \| **Number of responses** \| \| --- \| --- \| \| **Clinic related** \|  \| \| Relationship with colleagues/work politics \| 9 \| \| Patient expectations \| 6 \| \| Boss/supervisor/hospital expectations \| 5 \| \| Poor laboratory work \| 3 \| \| Poor clinic management \| 2 \| \| Dental Surgery Assistant (DSA)/DSA on MC/DSA poor performance \| 1/2/1 \| \| Change of DSA \| 1 \| \| Too many/long working hours \| 7 \| \| Career advancement/treatment of cases to learn \| 2 \| \| Pressure of meeting KPI \| 2 \| \| Treatment planning complex cases \| 2 \| \| Responsibility for advising patients \| 1 \| \| Incongruence between personal and company values \| 2 \| \| Non clinical duties/administration \| 3 \| \| Too many patients to see \| 1 \| \| Equipment failures/technical issues/inadequate equipment \| 6 \| \| Unable to get peaceful breaks \| 1 \| \| Time pressures \| 2 \| \| Poor staff training \| 1 \| \| Lack of work/building book/insufficient pts/pt flow \| 6 \| \| Clinic resources \| 1 \| \| **Patient-related** \|  \| \| Striving for the best for pts/personal expectations \| 1/2 \| \| Difficult/demanding patients \| 6 \| \| Complaints from patients \| 4 \| \| SG bureaucracy and rules/Singapore Dental Council \| 3 \| \| **Other** \|  \| \| Operator capability/execution of procedures/worry after procedure/not good enough \| 4 \| \| Legislation/law suit \| 1 \| \| Starting own practice whilst working \| 1 \| \| Unable to find a job \| 1 \| \| **Financial related** \|  \| \| Cost of CDE \| 1 \| \| Business overhead \| 2 \| \| Financial pressures – no salary when no work \| 2 \| \| Feeling guilty about going on holiday \| 1 \| \| **Physical/Mental** \|  \| \| Health/ Physical limitations – backache/Pain \| 5 \| \| Eyesight \| 3 \| \| Fear of personal physical pain \| 1 \| \| Sports related \| 1 \| \| Stress of a law suit \| 1 \| \| Loneliness \| 2 \| \| Ruminating over less than desirable outcomes \| 1 \| \| **Personal** \|  \| \| Family relationships/demands /and looking after young children \| 12 \| \| Loss of passion \| 1 \| \| Personal \| 4 \| \| Boredom/monotonous work \| 2/1 \| \| Quality of sleep \| 1 \| \| Work life balance \| 7 \| \| Housework \| 1 \|  \| **Other outcomes of stress (Q21)** \| **Number** \| \| --- \| --- \| \| **Clinical** \|  \| \| Missing something diagnostically \| 1 \| \| Impatient/short temper with new staff/staff/general \| 8 \| \| Patients not happy with treatment \| 1 \| \| Less job satisfaction \| 2 \| \| Treatment outcomes \| 1 \| \| Favoring less complex treatment \| 1 \| \| Substandard work (by own standard) \| 1 \| \| Pressure from work colleagues \| 1 \| \| Lack of time \| 1 \| \| Lack of time to write good clinical notes \| 1 \| \| Referring patients out \| 1 \| \| Staff treating patients poorly \| 1 \| \| **Patient-related** \|  \| \| Patient perception/satisfaction/expectation \| 2/2/2 \| \| Unable to meet patient expectation leading to tarnished reputation \| 1 \| \| Rushing procedures \| 3 \| \| Crying in front of a patient \| 1 \| \| Putting less effort/less energy with communication \| 2 \| \| Patient complaints \| 2 \| \| Unable to focus when treating patients \| 1 \| \| Getting irritated at patients \| 1 \| \| Making mistakes \| 2 \| \| **Physical/Mental** \|  \| \| Aching body \| 11 \| \| Exhaustion \| 2 \| \| Loss of patience with staff \| 2 \| \| Health issues \| 1 \| \| Overeating/weight \| 1/1 \| \| Feeling depressed/anxious \| 1/2 \| \| Substance use – alcohol/smoking \| 1 \| \| Affects sleep quality/getting to sleep \| 1/1 \| \| Venting to people near me \| 2 \| \| Pain due to spinal issues \| 1 \| \| Tired \| 4 \| \| Thinking about mistakes outside work \| 1 \| \| Feeling moody \| 2 \| \| Feeling numb \| 1 \| \| Feeling susceptible in the pandemic \| 1 \| \| Self-disappointment \| 4 \| \| Loss of appetite, feeling emotionally low & drained \| 1 \| \| **Financial** \|  \| \| Questioning by boss about low revenue \| 1 \| \| Low income \| 1 \| \| **Personal** \|  \| \| Feeling emotionally frustrated (with DSA) \| 1 \| \| No time for family/not emotionally present with family \| 3/1 \| \| Need a holiday \| 2 \| \| Affects interactions with family or friends outside work/venting \| 1/2 \| \| Not having energy for other interests/hobbies \| 1 \| \| Wanting to quit job \| 2 \| \| Tarnished reputation \| 1 \| | |
|  | |

| \| **Support for dentists with stress or burnout (Q23)** \| **Number** \| \| --- \| --- \| \|  \|  \| \| Support groups/counselling \| 43/21 \| \| Mentoring \| 5 \| \| Yoga, body posture, meditation, mindfulness/self care \| 6/1 \| \| Dental community \| 9 \| \| Sports/group activities or social events \| 13 \| \| Removing the stigma of setting work life boundaries and the unhealthy obsession with working more than those around you \| 1 \| \| Reduce stigma around seeking help/MH \| 4 \| \| Workshops that offers burnt out management strategies/MH \| 10/1 \| \| More support from the SDA \| 2 \| \| Patient education/public awareness about dentistry as a job \| 9 \| \| Media presentation of dentists, positive \| 4 \| \| Understanding family members and friends needs \| 1 \| \| Encourage breaks from work \| 1 \| \| Balance of number of patients \| 1 \| \| More support from DPL phone support/workshops \| 2 \| \| Have council and association that support dentists instead of regulating them all the time. \| 1 \| \| Provide more protection against litigation and public display of cases \| 2 \| \| SDC should make things clear on how could conditional registration dentists get their full license. Support with complaints \| 1 \| \| Support with complaints \| 3 \| \| Rest/holiday \| 7 \| \| Fewer working hours \| 11 \| \| More protection from SDA/SDC re patient complaints \| 1 \| \| Self regulation, management and empathy for self and modify situation \| 4 \| \| Lower own expectations \| 1 \| \| Reduced administration in government jobs \| 1 \| \| Reduced change of policies in government jobs \| 1 \| \| Empathy from colleagues \| 5 \| \| Change of environment \| 1 \| \| Help with staffing \| 1 \| \| Kindness \| 1 \| \| Guidelines \| 1 \| \| Address work life balance \| 6 \| \| Better remuneration \| 3 \| \| Better scheduling \| 1 \| \| Reduce government bureaucracy and legislation \| 2 \| \| Better selection of resilient students for dental school \| 1 \| \| Policies and rules to discourage defensive medicine /dentistry \| 1 \| \| Enough patients \| 1 \| \| Legislation to protect profession against unreasonable patients \| 1 \| \| Address uncertainty with SDC COC debate \| 1 \| |
| --- | --- | --- | --- | --- | --- | --- | --- | --- | --- | --- | --- | --- | --- | --- | --- | --- | --- | --- | --- | --- | --- | --- | --- | --- | --- | --- | --- | --- | --- | --- | --- | --- | --- | --- | --- | --- | --- | --- | --- | --- | --- | --- | --- | --- | --- | --- | --- | --- | --- | --- | --- | --- | --- | --- | --- | --- | --- | --- | --- | --- | --- | --- | --- | --- | --- | --- | --- | --- | --- | --- | --- | --- | --- | --- | --- | --- | --- | --- | --- | --- | --- | --- | --- | --- |
